# *Clostridium butyricum* enhances cognitive function in APP/PS1 mice by modulating neuropathology and regulating acetic acid levels in the gut microbiota

**DOI:** 10.1128/spectrum.00178-25

**Published:** 2025-07-07

**Authors:** Ye Shiqing, Lu Xinjie, Zhu Xiaotong, Chen Jiayan, Lu Jiahai, Chen Xiaodong, Lou Yongliang, Li Xiang

**Affiliations:** 1 Wenzhou Key Laboratory of Sanitary Microbiology, Wenzhou Medical University 214181, Wenzhou, Zhejiang, China; 2 Institute of One Health, Wenzhou Medical University 26453, Wenzhou, Zhejiang, China; 3 Key Laboratory of Laboratory Medicine, Ministry of Education, Wenzhou Medical University, Wenzhou, Zhejiang, China; 4 School of Laboratory Medicine and Life Sciences, Wenzhou Medical University, Wenzhou, Zhejiang, China; 5 Department of Neurology, Linyi Women & Children’s Healthcare Hospital, The First Affiliated Hospital of Shandong Medical College 721707 , Linyi, Shandong, China; MultiCare Health System, Tacoma, Washington, USA

**Keywords:** Alzheimer’s disease, *Clostridium butyricum*, intestinal microbiota, short-chain fatty acid, neuroinflammation

## Abstract

The amyloid deposition-to-cognitive impairment pathway is crucial in Alzheimer’s disease (AD) pathogenesis, with gut microbes influencing its development. Our study evaluated *Clostridium butyricum* MIYAIRI 588 (CBM588) as a potential AD treatment. In APP/PS1 mice, CBM588 improved cognitive function and alleviated colonic tissue pathology by modulating tight junction proteins (upregulating Claudin 1, ZO-1, and Occludin) and reducing inflammatory cytokines (IL-6, IL-1β, and TNF-α mRNA). It also reduced Aβ plaque deposition in the brain, regulated the JNK/CDK5/GSK-3β pathway to inhibit Tau hyperphosphorylation, and maintained neuronal integrity by downregulating Bax/Bcl-2 and inflammatory cytokines, thereby inhibiting apoptosis. While not altering the overall gut flora structure, CBM588 increased acetate-producing bacteria and decreased pro-inflammatory genera, reducing lipopolysaccharide levels. It also elevated short-chain fatty acids, particularly acetate, in colonic feces and brain tissue. Sodium acetate further inhibited BV2 cell apoptosis by suppressing JAK/STAT signaling and reducing Aβ and p-Tau expressions. In conclusion, CBM588 holds great potential as a novel probiotic for AD control.

**IMPORTANCE:**

The current study underscores the pivotal role of gut microbiota modulation in the treatment of Alzheimer’s disease(AD). Our comprehensive evaluation of CBM588 demonstrates its remarkable potential to ameliorate cognitive impairment in APP/PS1 mice by modulating gut microbiota composition, upregulating short-chain fatty acids, particularly acetate, and mitigating neuroinflammation. These findings not only provide novel insights into the gut-brain axis in AD but also offer a promising therapeutic strategy, highlighting the importance of targeting gut microbiota in future AD research and interventions.

## INTRODUCTION

Alzheimer’s disease (AD) is a primary degenerative brain disorder characterized by neuronal death (1) and is often associated with key neuropathological changes, including the progressive deposition of β-amyloid (Aβ) plaques and neural fibrillary tangles triggered by hyperphosphorylation of Tau protein (2). However, drugs targeting Aβ and Tau have had limited impact on disease progression (3), suggesting that AD is not solely a brain lesion disorder. The pathway from amyloid deposition to cognitive impairment is considered a cornerstone of AD pathogenesis, and studies have found that gut microbes are involved in the development of Aβ deposition pathology (4).

The gut microbiota contains over 100 trillion symbiotic microbial cells (5). It communicates bidirectionally with the brain through the microbiota-gut-brain axis (MGBA), connecting the gut and the nervous system to regulate behavior and brain immunity (6). Growing evidence ties gut microbiota imbalances to neurodegenerative diseases like AD (7). AD patients have less diverse gut flora than healthy individuals (8, 9), with fewer *Firmicutes* (probiotic bacteria) and more *Bacteroidetes* (pro-inflammatory bacteria) (8, 9). Gut microbes also produce amyloid and lipopolysaccharides that may drive AD-related inflammation (10, 11). These findings underscore the gut microbiota’s role in AD and its potential as a therapeutic target. Probiotics are considered one of the best preventive measures against cognitive decline in AD (12). Many *in vivo* studies and recent clinical trials have demonstrated the effectiveness of selected bacterial strains in slowing down AD progression. *Clostridium butyricum* is a probiotic that has been widely used for decades in the treatment of gastrointestinal disorders, cancer, and psychiatric disorders due to its safety, non-toxicity, and beneficial effects on host health (13–16). Additionally, *C. butyricum* has shown potential in studying brain-related diseases (17). Recently, a study in transgenic AD mice demonstrated that the use of *C. butyricum* attenuated the Aβ pathological load in the brains of APP/PS1 mice, accompanied by an increase in gut butyric acid levels. At the cellular level, butyric acid ameliorated neuroinflammation (18). Gut-derived butyric acid crosses the blood-brain barrier, inhibits neuroinflammation, and exerts neuroprotective effects (19). Therefore, CBM588 may be a candidate probiotic for improving cognitive impairment in Alzheimer’s disease.

Although the current literature suggests that *C. butyricum* has potential in AD prevention and treatment, the exact mechanisms behind these health benefits have not been fully elucidated. Most studies have shown changes in specific taxa of the intestinal flora after supplementation with *C. butyricum* (20), but the mechanisms behind this modulation remain unclear. Besides, while most studies suggest a causal effect of *C. butyricum* supplementation on short-chain fatty acid levels (21), direct evidence for this remains to be explored. Additionally, most studies based on probiotic interventions have been limited to changes in protein levels, with minimal focus on genetic differences, and have not elucidated the link between the gut and the brain, lacking a comprehensive view based on the MGBA.

This study will start with the intestinal flora of Alzheimer’s disease patients, analyzing changes in intestinal flora between AD patients and healthy human controls through 16S rDNA gene sequencing. Finally, this work will summarize the intestinal microbiota patterns of AD patients. CBM 588 was used to treat APP/PS1 double transgenic mice, and its effect on cognitive impairment in APP/PS1 mice was investigated using water maze experiments. The effect of CBM588 on the intestinal microbiota was analyzed by 16S rDNA sequencing. Targeted short-chain fatty acid (SCFA) assays were conducted on colonic fecal matter and mouse brain tissues to study CBM588 and SCFAs. The association between CBM588 and SCFAs was analyzed at the signaling pathway level, focusing on sodium acetate (NaAc)’s inhibition of neuropathology in BV2 cells. Protein- and gene-level studies further clarified how CBM588 improves cognitive impairment in APP/PS1 mice via the MGBA.

## MATERIALS AND METHODS

### Clinical participants and sample collection

Patients diagnosed with AD were recruited from specialized mental health clinics and inpatient facilities, whereas the healthy control group (HC) comprised individuals from health examination centers. Ethical approval for this study was granted by the Ethics Review Committee of Linyi Women & Children’s Healthcare Hospital and the First Affiliated Hospital of Shandong Medical College (approval number: KYL-YXLL-2021027). Following rigorous screening based on predefined recruitment criteria, 10 AD patients and 10 age- and gender-matched HC participants were enrolled. All participants were Han Chinese aged between 70 and 80 years, with comparable dietary habits, and residing in similar geographical areas. Participants had not consumed broad-spectrum antibiotics, probiotics, or probiotic preparations in the preceding 6 months. Individuals with severe organic diseases or concurrent mental disorders were excluded. Furthermore, all participants had intact gastrointestinal structures and had not undergone gastrointestinal surgery within the past 5 years. Fresh, formed stool samples were collected from each participant in the morning using sterile spoons to prevent environmental contamination. Three samples (200 mg each) were obtained from each individual, promptly frozen, and stored at −80°C to avoid cycles of freezing and thawing.

### Animal group distribution and treatment

Male B6C3-Tg (APPsw, PS1dE9) transgenic mice and age-matched C57BL/6JV wild type were acquired from Hangzhou ZiYuan Co., Ltd. at the age of 3 months. After a month-long isolation and quarantine period at the Laboratory Animal Center of Wenzhou Medical University, the mice were transferred to an SPF-grade controlled environment. The study protocol was approved by the Animal Experimentation Ethics Committee of Wenzhou Medical University (approval number: wydw2021-0394). At 4 months of age, APPswe, PSEN1dE9 (APP/PS1) double transgenic mice were randomly assigned to either the B6C3-Tg model group (Tg) or the CBM 588 treatment group (Tg + CB), with wild-type mice serving as the control group (WT) (*n* = 8 mice for each group). Mice in the Tg + CB group received daily gavage of CBM588 suspension at a dose of 0.2 mL per gavage (containing 2 × 10^8^ CFU/dose). Mice in the WT and Tg groups were gavaged daily with sterile phosphate-buffered saline (PBS) at a volume of 0.2 mL per gavage. After 4 weeks of continuous gavage, the Morris water maze (MWM) test was conducted to assess spatial learning and memory.

### Morris water maze test

Spatial learning and memory were evaluated using a circular MWM (120 cm diameter, 50 cm height) filled with white water maintained at 22°C (water depth: 30 cm) and surrounded by visual cues. The MWM was divided into four quadrants, with a submerged platform (6 cm diameter) located 1 cm below the water surface in the target quadrant (third quadrant). The MWM system automatically recorded latency, platform crossings, distance to the target quadrant, and swim path for each trial. During the 5-day training period, mice were introduced from four different quadrants to locate the submerged platform using extramaze cues. Each day consisted of four trials (60 s maximum duration) with a 5 min intertrial interval. The average of the four training durations per day was used to assess the learning performance. On the sixth day, the escape platform was removed, and mice entered the maze from the quadrant opposite to the target quadrant. Their swimming path and memory indicators were recorded within 60 s. Following the behavioral test, mice were euthanized, and samples (brain, colon, feces, and serum) were collected and stored at −80°C.

### Cell culture and treatment

BV2 microglia were obtained from ScienCell (USA) and cultured in high-glucose Dulbecco’s Modified Eagle’s Medium supplemented with 10% fetal bovine serum (Invitrogen, Waltham, MA), 100 units/mL penicillin, and 100 mg/mL streptomycin (Beyotime, Nanjing, China). Cells were maintained in an incubator with 5% CO_2_ at 37°C. After reaching optimal growth, BV2 cells were induced with 1 µg/mL lipopolysaccharide (LPS) to form the LPS group. LPS-induced inflammatory BV2 cells treated with a specific concentration of sodium acetate (NaAc) comprised the LPS + NaAc treatment group, while untreated BV2 cells served as the control group (Con). These groups were used for CCK8 assays, cell apoptosis assays, and western blot analysis, following the respective kit instructions.

### Fecal DNA extraction and 16S rDNA gene sequencing

Following the MWM test, fresh fecal samples were collected aseptically and immediately frozen at −80°C. DNA extraction was performed using a DNA extraction kit (QIAGEN, Hilden, Germany) according to the manufacturer’s instructions. The V3–V4 region of the 16S rDNA gene was amplified, and beta diversity was analyzed using UniFrac analysis. The 16S rDNA sequencing data were processed using QIIME version 1.9.0. Taxonomic information for each operational taxonomic unit (OTU) with 97% sequence similarity was obtained from the Greengenes database. OTUs with abundance values below 0.005% of the total sequenced amount were excluded. Linear discriminant analysis (LDA) of effect size (LEfSe) was used to analyze intestinal flora variability.

### Gas chromatography-mass spectrometry analysis of metabolites

Samples were placed in 1.5 mL centrifuge tubes containing 500 µL of water and 100 mg of glass beads. After mixing for 1 min, the samples were centrifuged at 12,000 rpm for 10 min. The supernatant was mixed with 100 µL of 15% phosphoric acid, 20 µL of 375 µg/mL internal standard solution (4-methylvaleric acid), and 280 µL of ether. The mixture was centrifuged at 4°C and 12,000 rpm for 10 min. The supernatant was analyzed using a Thermo Fisher Scientific Trace 1300 gas chromatograph equipped with an Agilent HP-INNOWAX capillary column (30 m × 0.25 mm ID × 0.25 µm). Helium was used as the carrier gas at a flow rate of 1.0 mL/min. The temperature program was as follows: initial temperature of 90°C increased to 120°C at 10°C/min, then to 150°C at 5°C/min, and finally to 250°C at 25°C/min for 2 min. Metabolites were detected using a Thermo ISQ 7000 mass spectrometer in electron impact ionization mode with an electron energy of 70 eV (18, 22). Calibration curves were constructed for the quantitative analysis.

### Hematoxylin and eosin staining

Colon tissue samples were embedded, sectioned into 5 μm-thick slices, dewaxed, rehydrated, stained with hematoxylin for 10 min, differentiated in hydrochloric acid ethanol for 30 s, stained with eosin for 10 s, rehydrated, and sealed with neutral gum. Histomorphological changes were observed microscopically (22).

### Congo red staining

Following behavioral testing, mice were anesthetized, perfused with PBS (pH 7.4), and fixed with 4% paraformaldehyde. Brain tissues were paraffin-embedded, sectioned into 5 μm-thick slices, stained with Congo red for 10 min, differentiated with alkaline solution for 10 s, counterstained with Lillie-Mayer hematoxylin for 2 , and evaluated microscopically for amyloid plaques.

### Western blot

Cerebral cortex samples were homogenized in RIPA lysis buffer, incubated on ice for 10 min, and centrifuged at 4°C at 12,000 ×*g* for 30 min. Protein concentrations were determined using the BCA Protein Assay Kit and adjusted accordingly. Proteins were separated by SDS-PAGE, transferred to membranes, blocked with 5% skim milk for 2 h, and incubated overnight at 4°C with the primary antibodies. The following primary antibodies were utilized: anti-ZO-1 (1:1,000, Diagbio, China), anti-Occludin (1:1,000, Diagbio, China), anti-Claudin 1 (1:1,000, Diagbio, China), anti-p-JNK (1:1,000, HUABIO, China), anti-JNK (1:1,000, HUABIO, China), anti-Tau (1:1,000, Diagbio, China), anti-p-Tau (Ser396) (1:1,000, Diagbio, China), anti-CDK5 (1:1,000, Diagbio, China), anti-p35p25 (1:1,000, HUABIO, China), anti-GSK3β (1:1,000, HUABIO, China), anti-p-GSK3β (1:1,000, HUABIO, China), anti-Bax (1:1,000, Diagbio, China), anti-Bcl-2 (1:1,000, Diagbio, China), anti-IL-6 (1:1,000, Diagbio, China), anti-p-JAK (1:1,000, Diagbio, China), anti-p-STAT3 (1:1,000, Diagbio, China), β-actin (1:3,000, Abways, China), and GAPDH (1:2,000, Diagbio, China). Membranes were washed, incubated with secondary antibodies (1:2,000, Beyotime, China) for 2 h at room temperature, and protein expression was detected using an ultrasensitive ECL chemiluminescence kit and a chemiluminescence imaging system. The grayscale intensity of the bands was quantified using ImageJ software, normalized against the corresponding internal controls, and subjected to statistical analysis via GraphPad Prism 7.0 software.

### Enzyme-linked immunosorbent assay

Reagents were equilibrated to room temperature, and standards were diluted in a gradient. Standards (50 µL) and samples (50 µL) were dispensed into their respective wells. A biotin-labeled antibody (50 µL) was added to all wells, which were then sealed to prevent evaporation. The plate was incubated at 37°C for 1 h. After incubation, the liquid was discarded, and the wells were washed three times with washing solution. Streptavidin-HRP (80 µL) was added to each well, followed by incubation at 37°C for 30 min. After discarding the reagents and washing the wells again, substrate was added and incubated at 37°C for 10 min. Finally, 50 µL of termination solution was added, and the optical density of each well was measured at 450 nm.

### RT-qPCR

RNA was extracted from tissue samples by grinding. The amount of starting RNA used for RT-qPCR was 2 µg. The extracted RNA was then reverse-transcribed into cDNA using a configured reaction system. For qPCR detection, the reaction system was programmed as follows: initial denaturation at 95°C for 30 s, followed by 40 cycles of denaturation at 95°C for 5 s and annealing/extension at 60°C for 60 s, with a dissociation stage at the end. Primer sequences used are listed in Table S1. The β-actin gene was used for internal normalized control. The relative mRNA expression of inflammatory factors was calculated by the 2^−ΔΔCt^ method.

### Preparation and ultrastructural observation of electron microscope samples

The brain tissue was fixed with 4% paraformaldehyde and dissected to obtain the hippocampal CA1 and CA3 regions, as well as the cortical tissue. The dissected tissue blocks were trimmed to 1 × 1 × 1.5 mm rectangular pieces and rinsed with 0.1 M PBS. They were then stored in 2.5% glutaraldehyde fixative solution at 4°C. After fixation, the blocks were rinsed with PBS three times and fixed in 1% osmium tetroxide at room temperature in the dark for 1 h. Following additional rinsing with double-distilled water, the samples were stained with uranyl acetate solution and subjected to progressive dehydration in acetone solutions. The samples were then infiltrated with acetone and epoxy resin-embedding agent in specific ratios, followed by baking at different temperatures to ensure proper polymerization. Semi- and ultra-thin sections were prepared, stained, and observed using transmission electron microscopy.

### RNA sequencing

Total RNA was extracted using TRIzol reagent, and its purity, concentration, and integrity were assessed using a spectrophotometer and a bioanalyzer. The transcriptome library was prepared using a specific RNA sequencing library prep kit, and paired-end (PE150) sequencing was conducted on the Illumina NovaSeq 6000 platform with an average sequencing depth of 40 million reads per sample. Low-quality reads (Q-score < 20) and adapters were removed using Trimmomatic, and PCR duplicates were marked and removed with Picard v2.18 (23). Clean reads were aligned to the human reference genome (GRCh38, Ensembl release 98) using STAR v2.7.9a (24) with default parameters, including splice junction detection and two-pass alignment mode. Gene expression levels were quantified as fragments per kilobase million based on the Ensembl annotation GTF file. Differential gene expression analysis was performed using DESeq2 with a significance threshold of |log2 fold change| > 1 and adjusted *P*-value < 0.05 (Benjamini-Hochberg correction). The Kyoto Encyclopedia of Genes and Genomes (KEGG) pathway enrichment analysis was conducted using clusterProfiler with a significance cutoff of *P*-value < 0.05.

### Cell apoptosis assay

Cells were washed, centrifuged, and resuspended in binding buffer. Apoptosis detection reagents (7-AAD and Annexin V-PE) were added to the cell suspension, and the mixture was incubated at room temperature. Apoptosis was analyzed using a flow cytometer, with data processed using specific software.

### Statistical analysis

Data are presented as mean ± SD and analyzed using GraphPad Prism 7.0 software. Differences between experimental groups were assessed using Student’s *t*-test, with the statistical significance set at *P* < 0.05.

## RESULTS

### Changes in gut microbiota of Alzheimer’s disease patients and healthy controls

**Fig 1:**
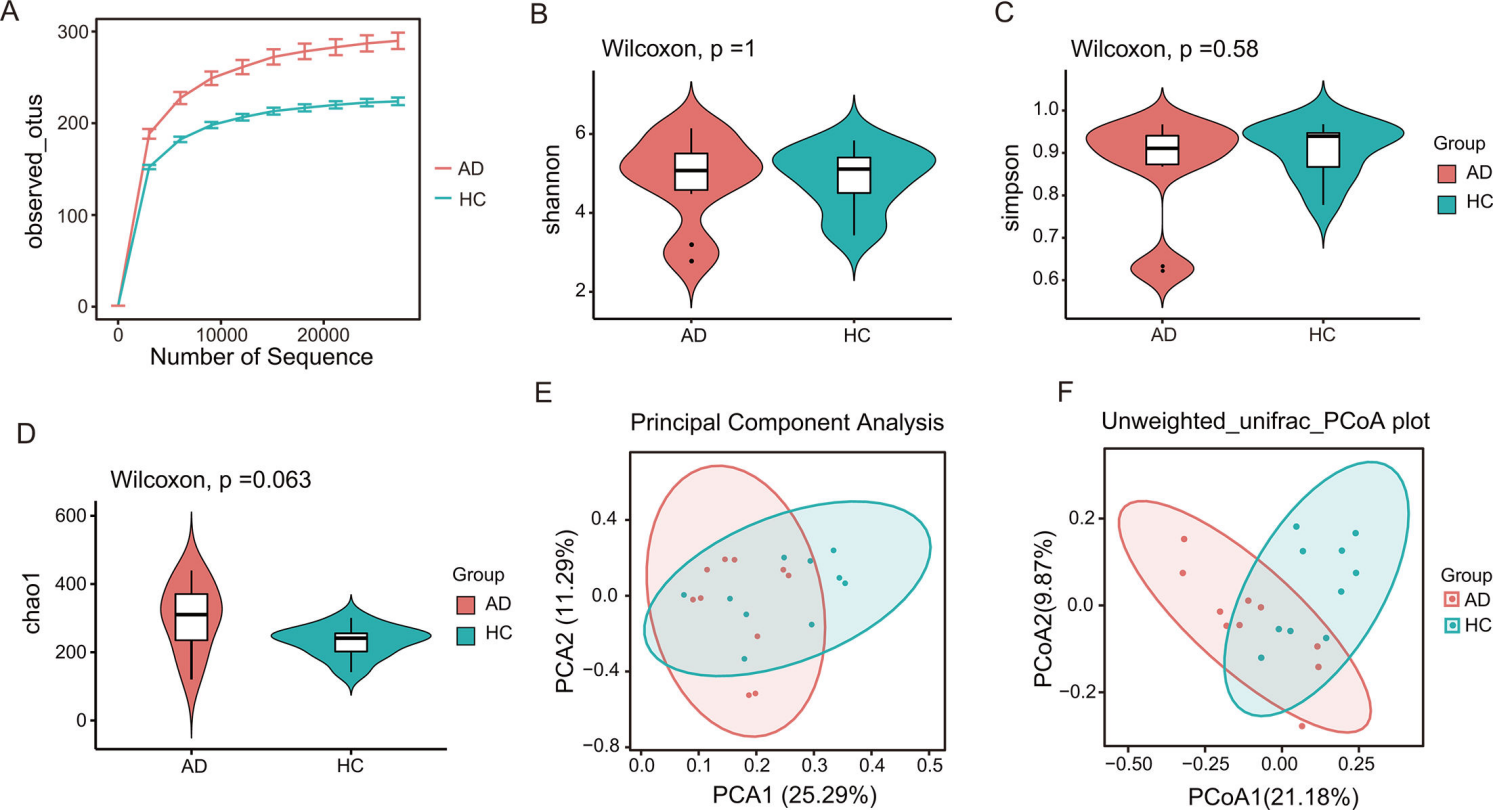
Comparison of the overall composition of the gut microbial community in the AD and HC groups. (**A**) Dilution curves and (**B**) Simpson’s index to assess species diversity. (**C**) Shannon’s index to assess community richness and evenness. (**D**) Chao1 is used to assess the number of species observed. (**E**) PCA: principal component analysis. (**F**) PCoA: principal coordinate analysis based on unweighted UniFrac distance. Each point in the figure represents a sample, and the distance corresponds to sample similarity. The red color represents the AD patient group, and the green color represents the HC group. *P < 0.05.

To explore the gut microbiota characteristics in AD patients in China, we collected fecal samples from age- and gender-matched AD patients and HC. 16S rDNA sequencing was performed to profile the gut microbiota. Dilution curves were used to test the reasonableness of the amount of data. The curves flattened out, indicating that they reflect the abundance of species in the samples (Fig. 1A). α-Diversity analysis revealed no significant differences in species richness or community homogeneity between AD patients and HC (*P* > 0.05) (Fig. 1B through D). β-Diversity analysis, which compares microbial community compositions, showed that the gut microbiota of AD patients and HCs partially overlapped but exhibited some segregation, indicating structural differences in the intestinal communities between the two groups (Fig. 1E and F).

### Differences in species abundance of gut microbiota between Alzheimer’s disease patients and healthy controls

**Fig 2:**
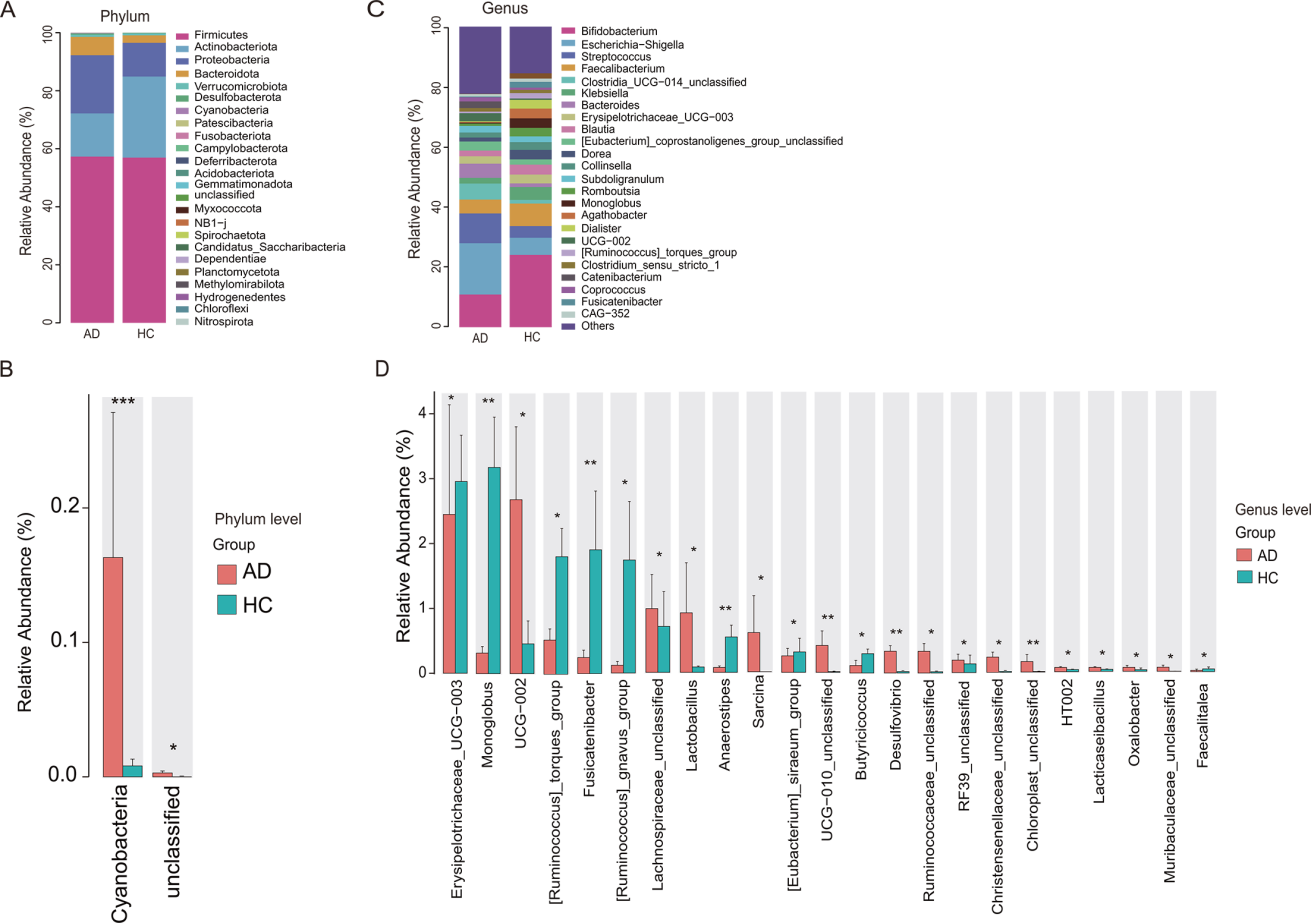
Clinical fecal gut microbiota relative abundance and significant differences at the phylum and genus levels. (**A**) Phyla ranked among the top 30 phyla in terms of relative abundance at the phylum level. (**B**) Phyla with significant differences at the phylum level. (**C**) Top 30 genera in terms of relative abundance at the genus level. (**D**) Bacterial abundances analyzed for significant differences at the genus level were all greater than 0.01%. The red color represents the AD patient group, and the green color represents the HC group. *P < 0.05; **P < 0.01; ***P < 0.001.

We analyzed the top 30 abundant species, classifying them at the phylum and genus levels using amplicon sequence variant abundance and annotation tables. At the phylum level (Fig. 2A), the relative abundance of *Bacteroidota* was higher in the AD group compared to the HC group (6.32% vs. 2.56%), while that of *Actinobacteriota* was lower in the AD group (14.87% vs. 27.92%). Additionally, the relative abundance of *Cyanobacteria* was significantly higher in the AD group (*P* < 0.01) (Fig. 2B).

At the genus level (Fig. 2C and D), the gut microbiota of AD patients showed an increased abundance of pro-inflammatory genera, including *Escherichia-Shigella* (17.06% vs. 5.68%), *Streptococcus* (9.92% vs. 3.89%), *Bacteroides* (4.76% vs. 1.19%), *Saricin*a (*P* < 0.05), and *Desulfovibrio* (*P* < 0.01). Conversely, some short-chain fatty acid-producing genera, such as *Bifidobacterium* (10.88% vs. 24.08%), *Faecalibacterium* (4.59% vs. 7.50%), *Dorea* (1.33% vs. 3.15%), *[Ruminococcus]_torques*_group (*P* < 0.05), *Fusicatenibacter* (*P* < 0.01), *Anaerostipes* (*P* < 0.01), and *Butyricicoccus* (*P* < 0.05), were significantly reduced in abundance. These findings suggest that a reduction in short-chain fatty acid-producing bacteria may contribute to the development and progression of AD.

### CBM588 improves cognitive impairment in Tg mice

**Fig 3:**
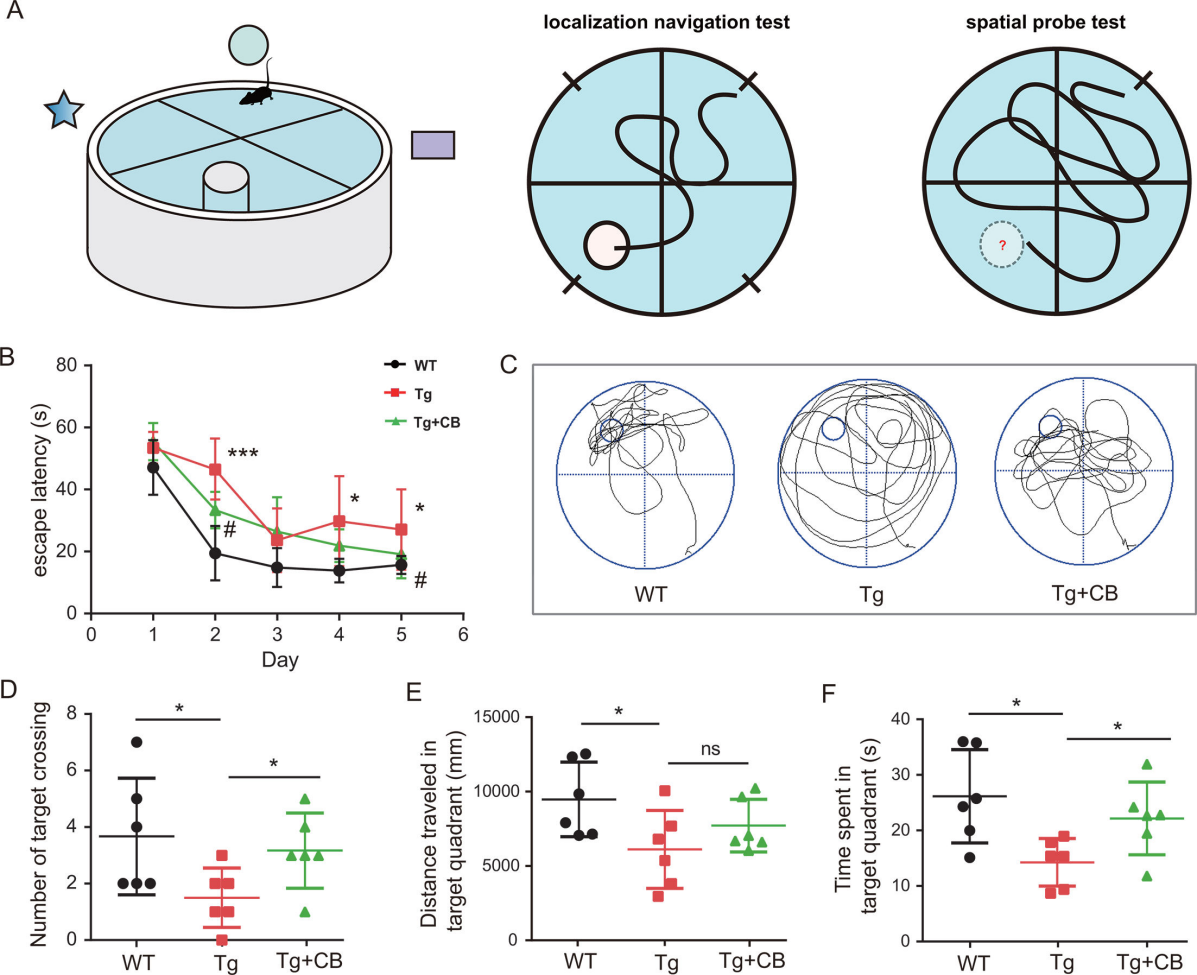
Effects of CBM588 on cognitive deficits in APP/PS1 mice. (**A**) Experimental protocol for MWM. Localization navigation test: five consecutive days of training, recording the time for the mice to locate the platforms from the four quadrants of the water entry point; and spatial probe test: on day 6, the platform was removed, and the mice entered the water from the quadrant on the opposite side of the original platform quadrant. The number of times the mice crossed the platform, the time in the target quadrant, and the distance traveled to the target quadrant were recorded (*n* = 6 for each group). (**B**) Latency of mouse avoidance. (**C**) Representative swimming routes in the platform-free exploration period of the MWM test. (**D**) Number of times the mouse crossed the platform. (**E**) Distance traveled by the mouse in the target quadrant. (**F**) Time spent by the mouse in the target quadrant. Black circles represent the WT group; red squares represent the Tg group; and green triangles represent the Tg + CB group. Data were analyzed using one-way ANOVA, followed by Dunnett’s multiple-comparison test (CDE) and repeated-measures ANOVA, followed by Tukey’s multiple-comparison test (**A**). Data were expressed as mean ± SD. # Tg vs Tg + CB; * Tg vs WT. *P < 0.05, ***P < 0.001; #*P* < 0.05.

CBM588 is a probiotic preparation containing live *C. butyricum* (CB), a strain known for producing various short-chain fatty acids, predominantly butyric acid (Fig. S1). The MWM experiment was utilized to assess the impact of CBM588 on cognitive impairment in C57BL/6JV (WT) and B6C3-Tg (Tg) mice. In the localization navigation test, the Tg group exhibited a longer escape latency compared to the WT group (day 2: *P* < 0.001; days 4 and 5: *P* < 0.05) (Fig. 3B), indicating a spatial learning deficit. However, the Tg + CB group showed a significantly shorter escape latency (day 2: *P* < 0.05; day 5: *P* < 0.05) compared to the Tg group (Fig. 3B), suggesting that CBM588 improves spatial learning ability in Tg mice. In the spatial exploration test, the Tg group displayed significantly fewer platform crossings (*P* < 0.05), less time spent in the target quadrant (*P* < 0.05), and reduced distance traveled in the target quadrant (*P* < 0.05) compared to the WT group (Fig. 3D through F). In contrast, the Tg + CB group showed a significant increase in the number of platform crossings (*P* < 0.05) and time spent in the target quadrant (*P* < 0.05) compared to the Tg group, indicating that CBM588 alleviates spatial learning deficits in these mice.

### CBM588 suppresses intestinal inflammation by regulating intestinal barrier function and reducing serum LPS levels in Tg mice

**Fig 4:**
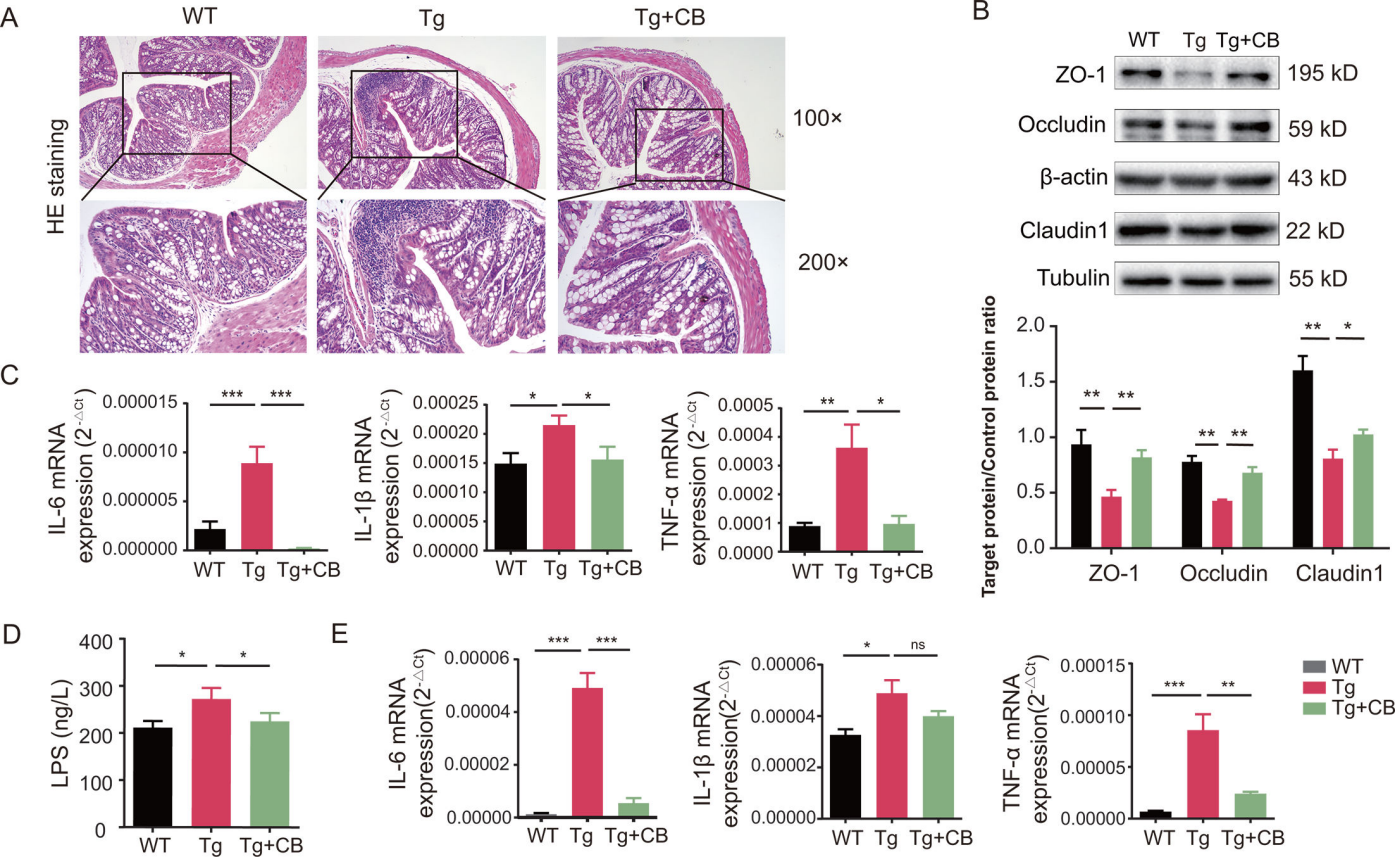
Effects of CBM588 on inflammatory factors in the colon and brain tissues of mice in the Tg group. (**A**) HE staining of mouse colon tissues with 100× and 200× magnifications. (**B**) Expression level of barrier-related proteins in mouse colon tissues (*n* = 3 for each group). (**C**) mRNA expression of inflammatory factors in mouse colon tissues (*n* = 3 for each group). (**D**) Serum LPS levels in mice (*n* = 3 for each group). (**E**) mRNA expression of IL-6, IL-1β, and TNF-α in mouse brain tissues (*n* = 3 for each group). All data were analyzed by one-way ANOVA, followed by Tukey’s multiple-comparison test. Data are expressed as mean ± SD. *P < 0.05; **P < 0.01; ***P < 0.001.

To further evaluate the effects of CBM588 gavage on the intestinal tract, hematoxylin and eosin staining was performed on colonic tissues from each group. As shown in Fig. 4A, the WT group exhibited intact and clear colonic mucosal structure with regularly arranged glandular structures in the lamina propria. In contrast, the Tg group displayed impaired colonic barriers, including edema of colonic tissues, thinning of the submucosal layer and muscularis propria, and extensive inflammatory cell infiltration. The Tg + CB group showed significant improvement in the colonic barrier morphology and reduced inflammatory cell infiltration, suggesting that CBM588 improves the colonic barrier pathology in Tg mice.

To investigate the molecular mechanisms underlying these observations, we examined barrier-related protein expression using western blotting. Figure 4B demonstrates that the expression levels of barrier-associated proteins Claudin-1, ZO-1, and Occludin were significantly lower in the Tg group compared to the WT group (*P* < 0.01). Conversely, the expression levels of these barrier proteins were significantly higher in the Tg + CB group compared to the Tg group (*P* < 0.01, *P* < 0.01, *P* < 0.05, respectively), indicating that CBM588 improves the colonic barrier integrity in Tg mice by upregulating barrier-associated proteins.

Additionally, to explore the gene-level differences in colonic inflammatory cell infiltration, we analyzed the expression levels of colonic inflammatory factors by RT-qPCR. As shown in Fig. 4C, the relative mRNA expression levels of IL-6, TNF-α, and IL-1β were significantly higher in the Tg group compared to the WT group (*P* < 0.001, *P* < 0.01, and *P* < 0.05, respectively), reflecting inflammation in the intestines of Tg mice. In contrast, the CBM588 intervention (Tg + CB) significantly reduced the expression of these inflammatory factors (*P* < 0.001, *P* < 0.05, *P* < 0.05, respectively), suggesting that CBM588 effectively alleviates colonic inflammation in APP/PS1 double transgenic mice.

Besides, serum LPS levels were measured using enzyme-linked immunosorbent assay (Fig. 4D). The results revealed that serum LPS levels in the Tg group were significantly higher than those in the WT group (*P* < 0.05). However, CBM588 intervention notably reduced serum LPS levels (*P* < 0.001). Additionally, as shown in Fig. 4E, mRNA expression levels of IL-6, TNF-α, and IL-1β in the brain tissues of the Tg group were elevated compared to those in the WT group (*P* < 0.001, *P* < 0.001, *P* < 0.05, respectively). In contrast, the levels of IL-6 and TNF-α were significantly lower in the Tg + CB group (*P* < 0.001, *P* < 0.01, respectively), indicating that *C. butyricum* may mitigate brain tissue inflammation by reducing LPS levels in Tg mice.

### CBM588 improves β-amyloid deposition and Tau hyperphosphorylation in the brains of Tg mice

**Fig 5:**
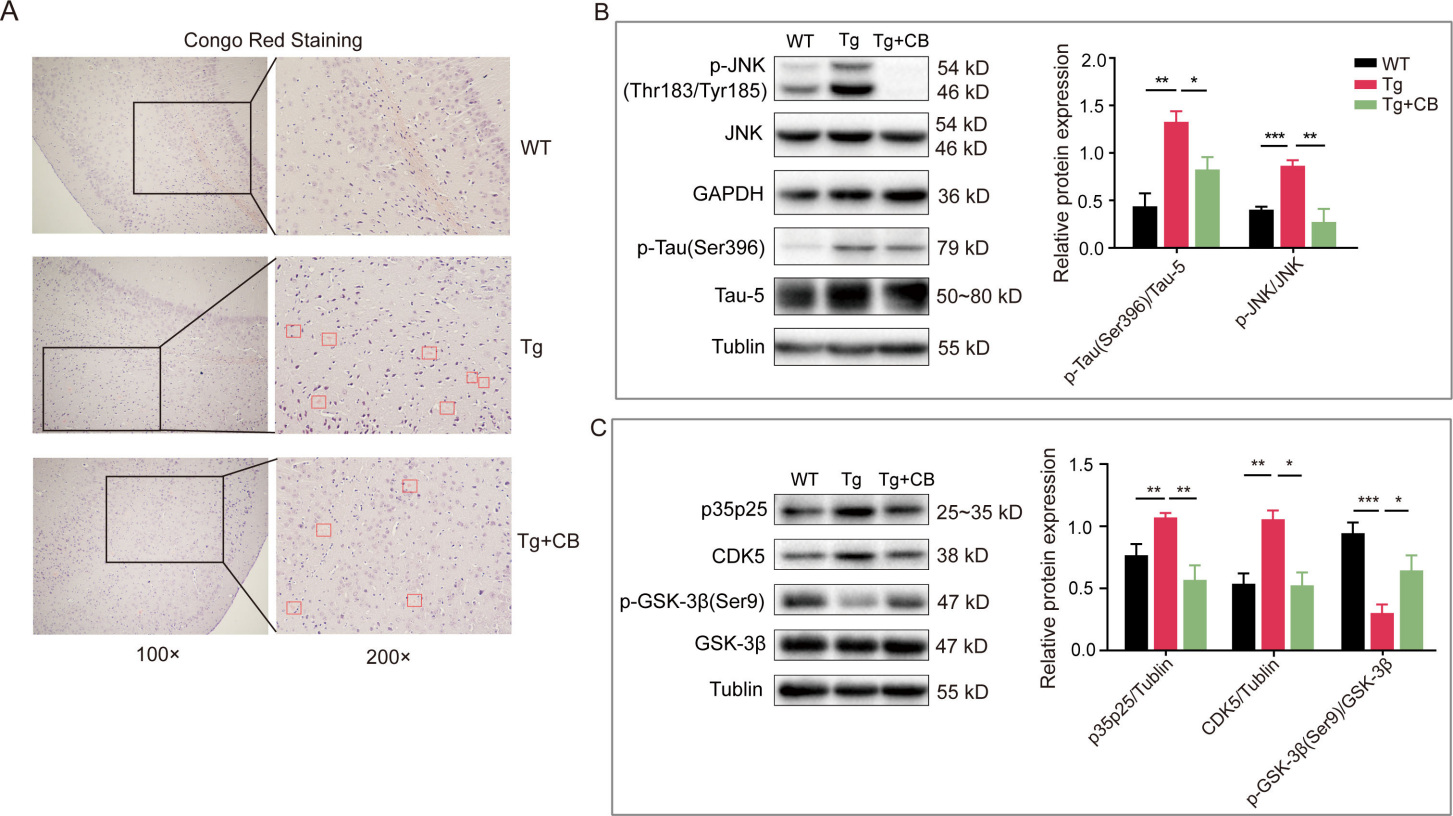
Effect of CBM588 on the brain pathological features of APP/PS1 mice. (**A**) Congo red staining; boxes represent 200× magnified areas, and arrows indicate amyloid plaques positive for Congo red staining (as red plaques). (**B and C**) Protein expression levels of Tau, p-Tau, JNK, p-JNK, p-GSK-3b (Ser9), GSK-3β, CDK5, and p35p25 in the mouse brain tissues (*n* = 3 for each group). All data were analyzed by one-way ANOVA, followed by Tukey’s multiple-comparison test. Data are expressed as mean ± SD. *P < 0.05; **P < 0.01; ***P < 0.001.

The two major pathological features of AD are Aβ deposition and Tau protein hyperphosphorylation. Congo red staining showed substantial amyloid plaque deposition in the brains of Tg mice, whereas Aβ levels were reduced in the brains of mice in the Tg + CB group (Fig. 5A), indicating that CBM588 attenuated the amyloid deposition in Tg mice.

JNK, GSK-3β, and CDK5 are key kinases involved in Tau phosphorylation. The upregulation of these proteins can significantly affect Tau phosphorylation and promote the progression of Alzheimer’s disease (25–27). Besides, p35p25 could bind to CDK5, and their complex further promotes Tau phosphorylation (28). The western blot analysis showed that the ratios of p-Tau(Ser396)/Tau-5 and p-JNK/JNK were significantly increased in the Tg group compared to the WT group (*P* < 0.01, *P* < 0.001), but these were significantly reduced in the Tg + CB group (*P* < 0.05, *P* < 0.01) (Fig. 5B). Furthermore, the expression level of CDK5 protein was decreased, and the phosphorylation level of GSK-3β was increased in the Tg + CB group compared to the Tg group in relation to the downregulation of p35p25 and the upregulation of the Ser9 phosphorylation site of GSK-3β, respectively (Fig. 5C). These results suggest that CBM588 inhibits Tau overphosphorylation by regulating the JNK/CDK5/GSK-3β signaling pathway, thereby ameliorating the pathological features in Tg mice.

### CBM588 inhibits neuronal cell apoptosis in Tg mice

**Fig 6:**
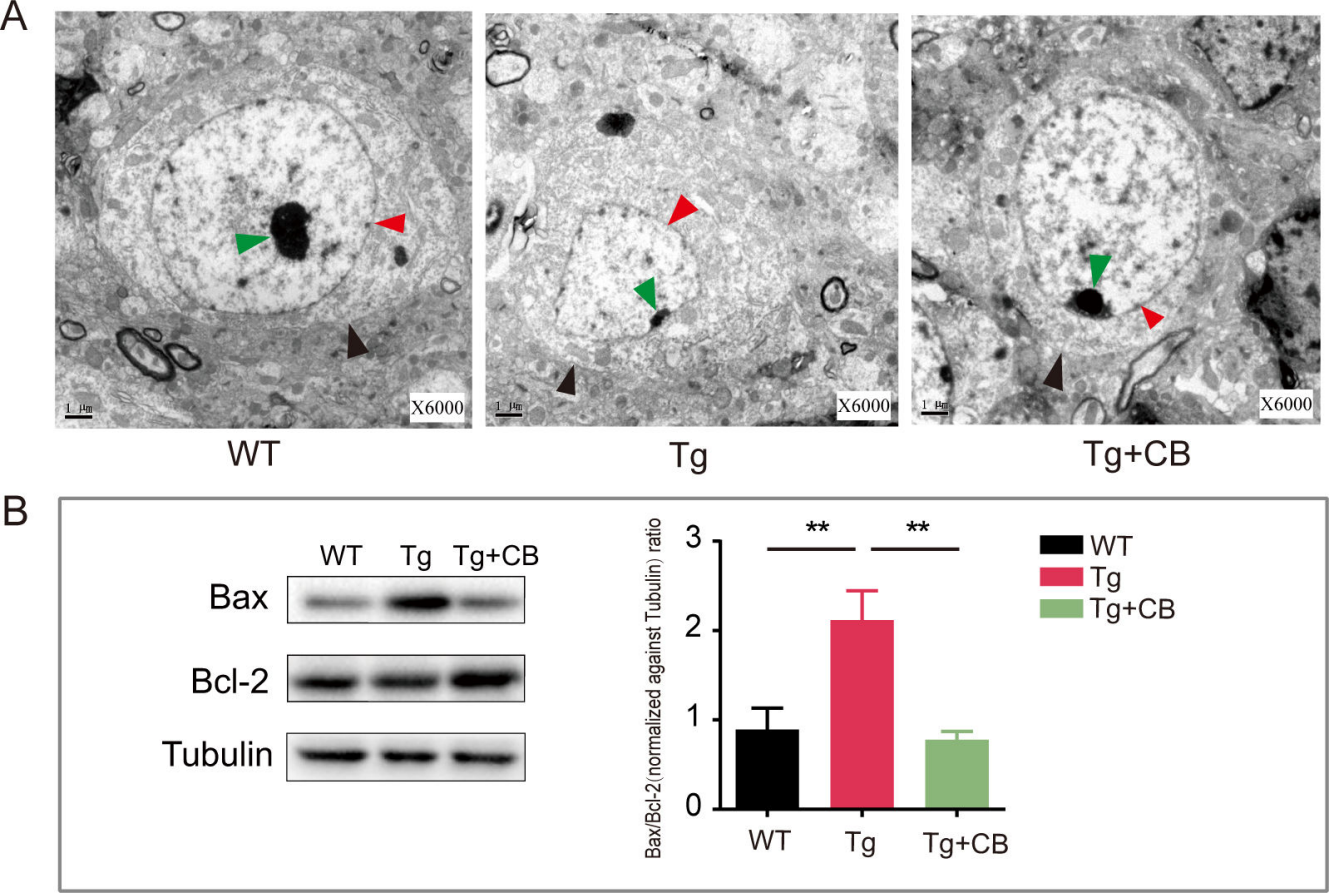
Effect of CBM588 on apoptosis of brain neuronal cells in APP/PS1 mice. (**A**) Transmission electron microscopy observation of neuronal morphology in mouse brain tissues. The black arrow points to the cell membrane; the red arrow points to the nuclear membrane; and the green arrow points to the nucleolus. (**B**) WB detection of Bax and Bcl-2 protein expression levels in mouse brain tissues (*n* = 3 for each group). All data were analyzed by one-way ANOVA, followed by Tukey’s multiple-comparison test. Data are expressed as mean ± SD. **P < 0.01.

Neuronal apoptosis is a significant factor in AD. Electron microscopy of mouse hippocampal tissues revealed that neurons in the WT group had a rounded morphology with intact nuclei, while neurons in the Tg group showed abnormal shapes with displaced and atrophied nuclei, indicating apoptosis. In contrast, neuronal pathology in the Tg + CB group was significantly improved, suggesting that CBM588 can reduce neuronal apoptosis in Tg mice (Fig. 6A). The western blot analysis of apoptosis-related proteins (Fig. 6B) revealed that the Bax/Bcl-2 ratio was significantly higher in the Tg group compared to the WT group (*P* < 0.05). The Bax/Bcl-2 ratio in the Tg + CB group was reduced, indicating that CBM588 could inhibit neuronal apoptosis to some extent.

### CBM588 alters the composition of gut microbiota in Tg mice

**Fig 7:**
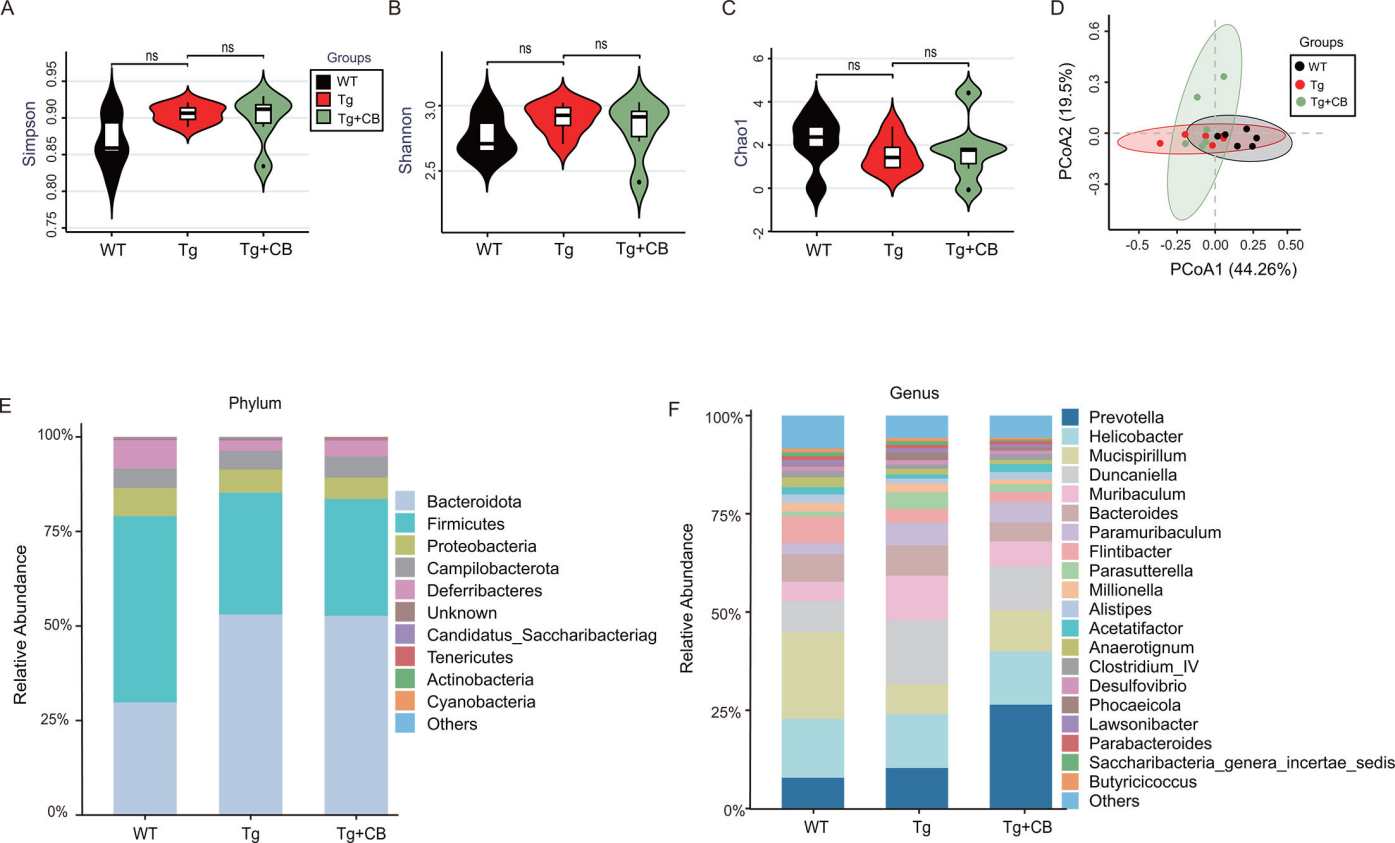
Effect of CBM588 on the structure of intestinal microbiota in APP/PS1 mice. (**A–C**) α-Diversity assessment indices: Chao1, Shannon, and Simpson. (**D**) β-Diversity analysis: PCoA showed that there was no significant difference between the three groups of mice in the structure of intestinal flora. (**E**) Histogram of the relative abundance at the phylum level of mouse intestinal flora, showing the top 10 phyla in relative abundance among the three groups. (**F**) Histogram of the relative abundance at the genus level of mouse intestinal flora, showing the top 20 phyla in relative abundance. ns, *P* > 0.05.

The effects of CBM588 on the gut microbiota in Tg mice were evaluated using 16S rDNA sequencing. α-Diversity results showed no significant difference in species diversity among the three groups (Fig. 7A through C). However, β-diversity results indicated differences in the overall structure of gut microbiota among the groups, particularly in the abundance of specific bacteria at the phylum and genus levels (Fig. 7D).

The species composition analysis revealed that, at the phylum level (Fig. 7E), the relative abundance of *Bacteroidota*, which includes many pathogenic bacteria, was higher in the Tg group than in the WT group (53.00% vs. 29.78%), while that of *Firmicutes*, which include many beneficial bacteria, was lower in the Tg group (32.25% vs. 49.25%). After the CBM588 intervention, the relative abundances of *Bacteroidota* and *Firmicutes* were slightly different between the Tg + CB and Tg groups (52.70% vs 53.00% and 30.87% vs 32.25%).

At the genus level (Fig. 7F), the abundance of pro-inflammatory genera, such as *Duncaniella*, *Bacteroides*, and *Parasutterella* (2.74% vs. 5.88%, 2.37% vs. 2.77%, 0.43% vs. 1.52%), increased in the Tg group. Conversely, the abundance of short-chain fatty acid-producing genera, including *Alistipes*, *Acetatifactor*, *Parabacteroides*, and *Butyricicoccus*, decreased. Notably, after the CBM588 intervention, the abundance of pro-inflammatory bacteria decreased, while that of acetic acid-producing bacteria, such as *Alistipes* and *Acetatifactor*, increased significantly. These findings suggest that CBM588 alters the gut microbiota composition in Tg mice by downregulating pro-inflammatory bacteria and upregulating short-chain fatty acid-producing bacteria.

**Fig 8:**
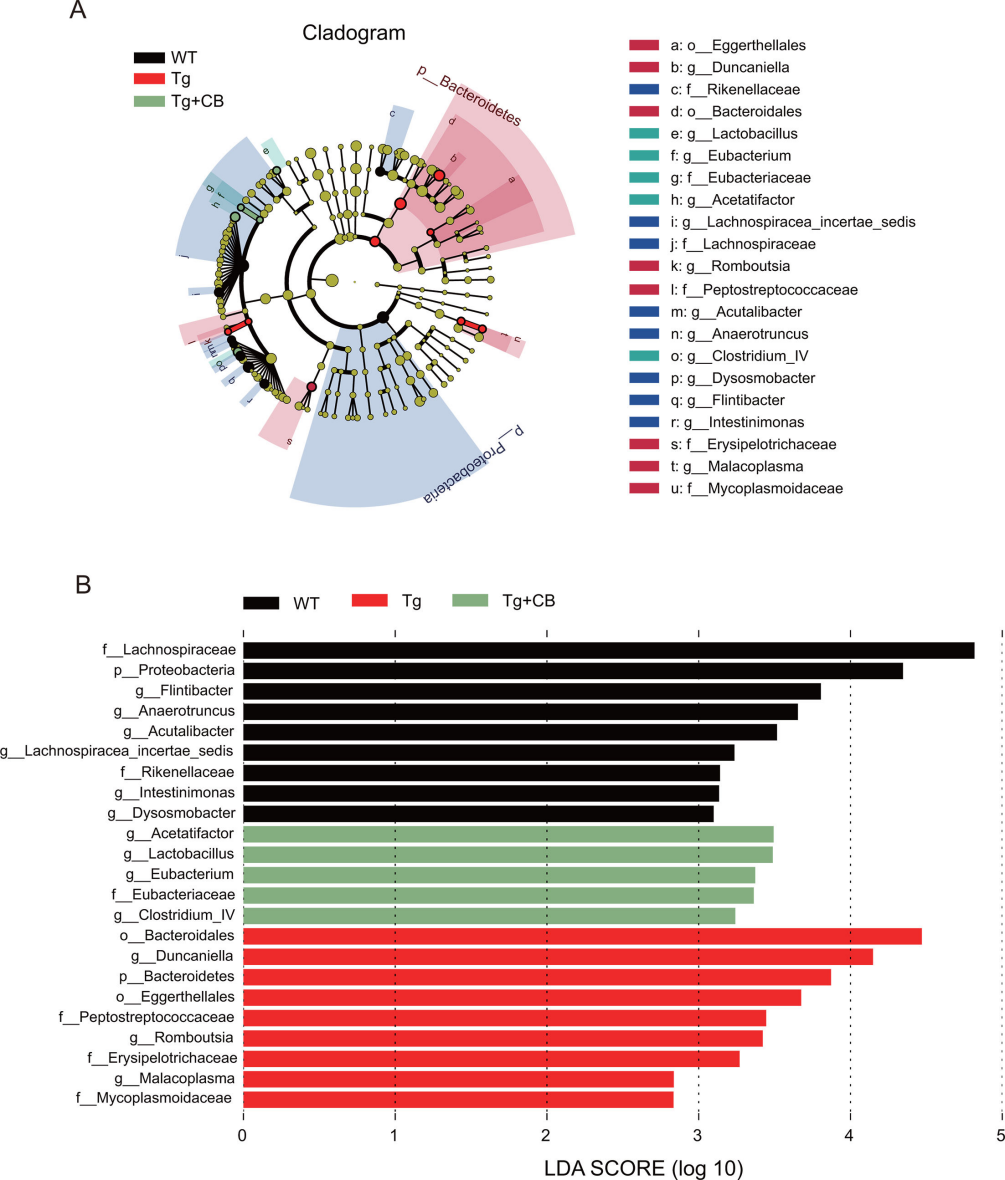
LEfSe analysis of the intestinal flora of mice in each group. (**A**) Cladogram: to show the taxonomic hierarchical distribution of marker species that were significantly enriched in each group of community samples. (**B**) LDA bar graph: to show the species that were significantly enriched and their degree of importance within each group. The length of the bar graph corresponds to the LDA value, and the larger the LDA value, the more significant the difference.

Gut microbial differences between groups were further assessed using LEfSe. As shown in Fig. 8A and B, the abundance of pro-inflammatory bacteria, such as *Bacteroidetes*, *Duncaniella*, *Peptostreptococcaceae*, *Romboutsia*, and *Erysipelotrichaceae*, increased in the Tg group, most of which are gram-negative, potentially explaining the increased intestinal inflammation and serum LPS levels in Tg mice. In contrast, the abundance of short-chain fatty acid-producing bacteria, especially *Acetatifactor*, *Lactobacillus*, and *Eubacterium*, increased significantly in the Tg + CB group after CBM588 intervention. This suggests that the improvement of cognitive deficits in Tg mice by CBM588 may involve an increase in acetic acid levels.

### *Clostridium butyricum* upregulates levels of short-chain fatty acids, primarily acetate, in the feces and brain tissue of Tg mice

**Fig 9:**
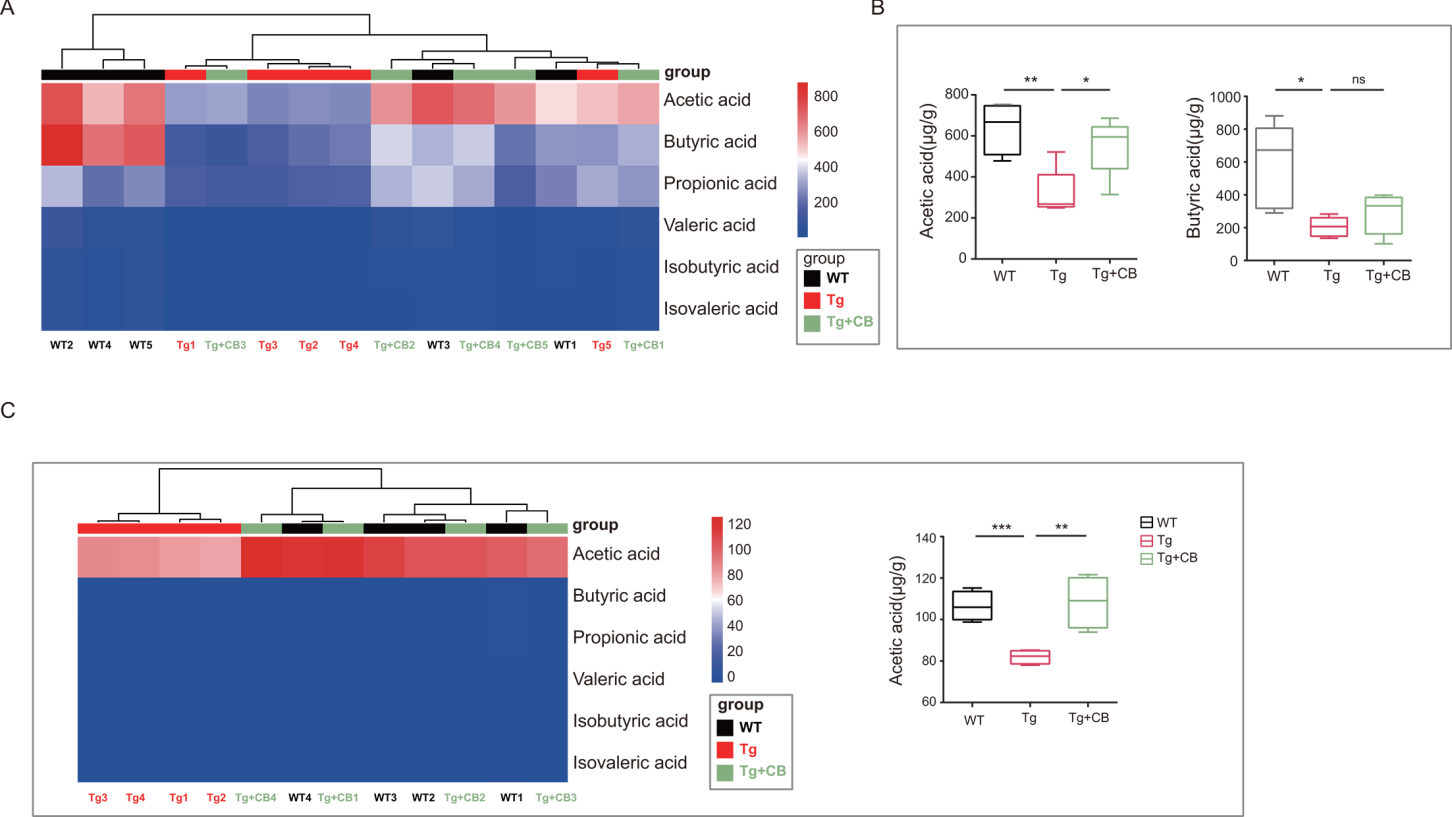
Analysis of targeted short-chain fatty acids in mouse intestinal contents. (**A**) Heatmap of the mouse intestinal short-chain fatty acid content and clustering of mice based on the short-chain fatty acid yield. Red indicates more content; blue indicates less content; and color shades indicate the degree. (**B**) Significant difference analysis of several common short-chain fatty acids in three groups of mice: acetic acid, propionic acid, butyrate, valeric acid, isobutyric acid, and isovaleric acid. (**C**) Heatmap of the short-chain fatty acid content in mouse brain tissues and clustering of mice based on the short-chain fatty acid yield. Red indicates more content; blue indicates less content; and color shades indicate the degree; Significant difference analysis of the acetic acid content in mouse brain tissues. *P < 0.05; **P < 0.01; ***P < 0.001.

Given that CBM588 intervention impacted the abundance of SCFA-producing bacteria in the gut microbiota of Tg mice, we performed microbial metabolite analyses targeting SCFAs in colonic contents (Fig. 9A). The results showed that the levels of several SCFAs were significantly lower in the Tg group compared to the WT group. In contrast, SCFA levels, particularly acetic acid, increased significantly in the Tg + CB group (*P* < 0.05) (Fig. 9B), indicating that CBM588 intervention enhanced SCFA production with a notable increase in acetic acid.

Since SCFAs can cross the blood-brain barrier, we investigated SCFA levels in the brain tissues. Targeted assays revealed that the acetic acid content was significantly lower in the Tg group compared to the WT group (*P* < 0.001), while acetic acid levels were significantly increased in the Tg + CB group (*P* < 0.01) (Fig. 9C). These results suggest that CBM588 upregulates SCFA levels, especially acetic acid, in the brain tissue, indicating its potential involvement in AD pathology.

### Acetate improves cognitive impairment in Tg mice through immune-related pathways in the brain

**Fig 10:**
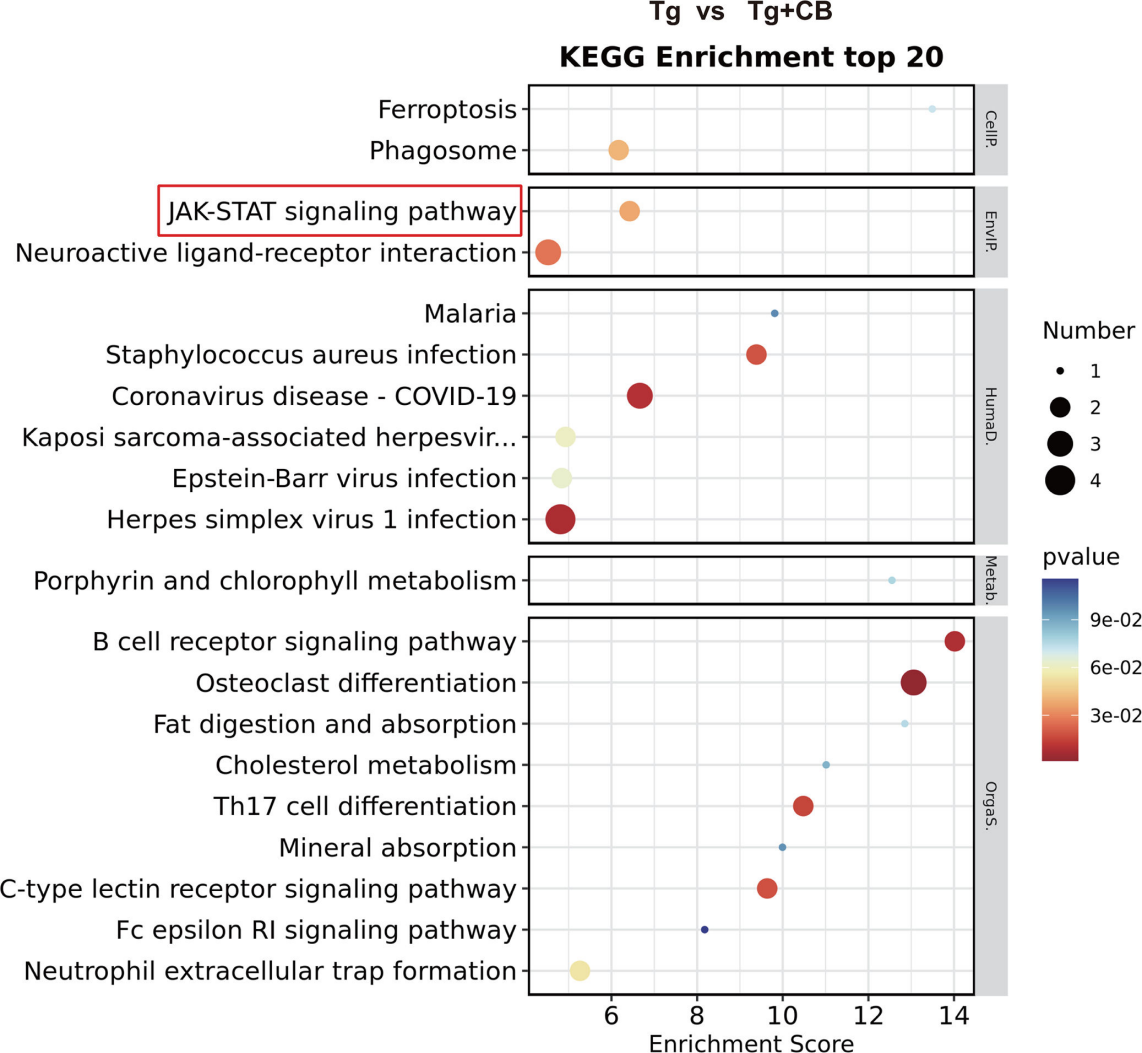
KEGG pathway enrichment analysis of differentially expressed genes in brain transcriptome (Tg vs. Tg + CB). The vertical axis is the pathway, and the horizontal axis is the enrichment score. The larger the bubble, the higher the number of differentially encoded genes, and the color of the bubble changes from blue to white to yellow to red. The color of the bubble indicates the *P*-value of the pathway. The smaller the *P*-value, the redder the bubble colo and the more significant the pathway enrichment. Generally speaking, the redder and larger the bubbles are, the more important the pathway is. The more to the right of the bubble, the more important the pathway is. The gray area of the pathway indicates the functional classification of the pathway entry at level 1. There are six major classifications at level 1: cellular processes, environmental information processing, genetic information, human diseases, metabolism, and organismal systems.

Pathway analysis of differential gene expression using the KEGG database identified key pathways enriched in Tg mice after CBM588 intervention (Fig. 10). DEGs were primarily enriched in cellular processes, environmental information processing, human diseases, metabolism, and organismal systems. Notably, pathways related to bacterial, viral, and parasitic infections, as well as porphyrin and chlorophyll metabolism, were significantly enriched. Among the immune-related pathways, B-cell receptor signaling, Th17 cell differentiation, and C-type lectin receptor signaling were highlighted, suggesting that acetic acid derived from CBM588 intervention may modulate immune system pathways in the brain, potentially ameliorating cognitive deficits in Tg mice. Additionally, pathways related to the digestive system, such as fat digestion and absorption, cholesterol metabolism, and mineral absorption, were also enriched. The JAK-STAT signaling pathway related to environmental information processing was notably enriched, indicating its potential role in the AD pathology.

### Sodium acetate inhibits LPS-induced apoptosis in BV2 cells

**Fig 11:**
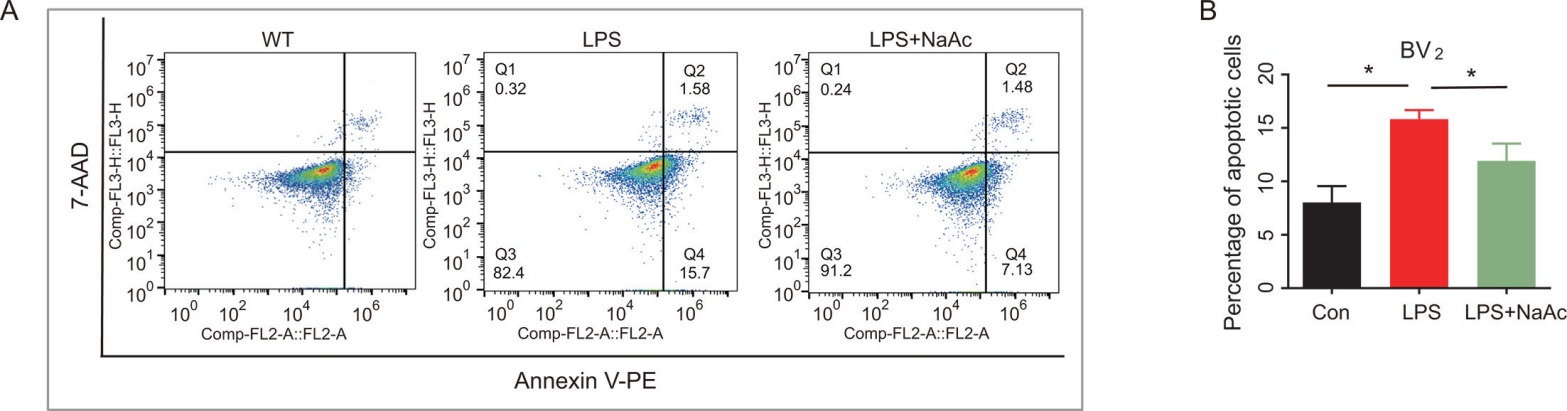
NaAc decreased apoptosis in BV2 cells with LPS-induced inflammation. (**A**) Flow cytometry to detect the effect of NaAc on microglia. Q1: the cells in this region were necrotic cells, and there may be a few late apoptotic cells, mechanically damaged cells; Q2: the cells in this region were late apoptotic cells; Q3: the cells in this region were early apoptotic cells; and Q4: the cells in this region were live fine. (**B**) Statistical graphs were recorded as the apoptotic rate of this cell with Q2 + Q3 (*n* = 3 for each group). All data were analyzed by one-way ANOVA, followed by Tukey’s multiple-comparison test. Data are expressed as mean ± SD. *P < 0.05.

BV2 cells were treated to evaluate the effect of sodium acetate (NaAc) on LPS-induced apoptosis. Flow cytometry data (Fig. 11) showed a significant increase in both early and late apoptosis rates in BV2 cells induced by LPS, indicating LPS-induced apoptosis. However, sodium acetate treatment significantly reduced both early and late apoptosis rates, suggesting that sodium acetate can effectively suppress apoptosis in BV2 cells.

### Sodium acetate improves AD pathology in BV2 cells by inhibiting the IL-6-Induced JAK/STAT3 signaling pathway

**Fig 12:**
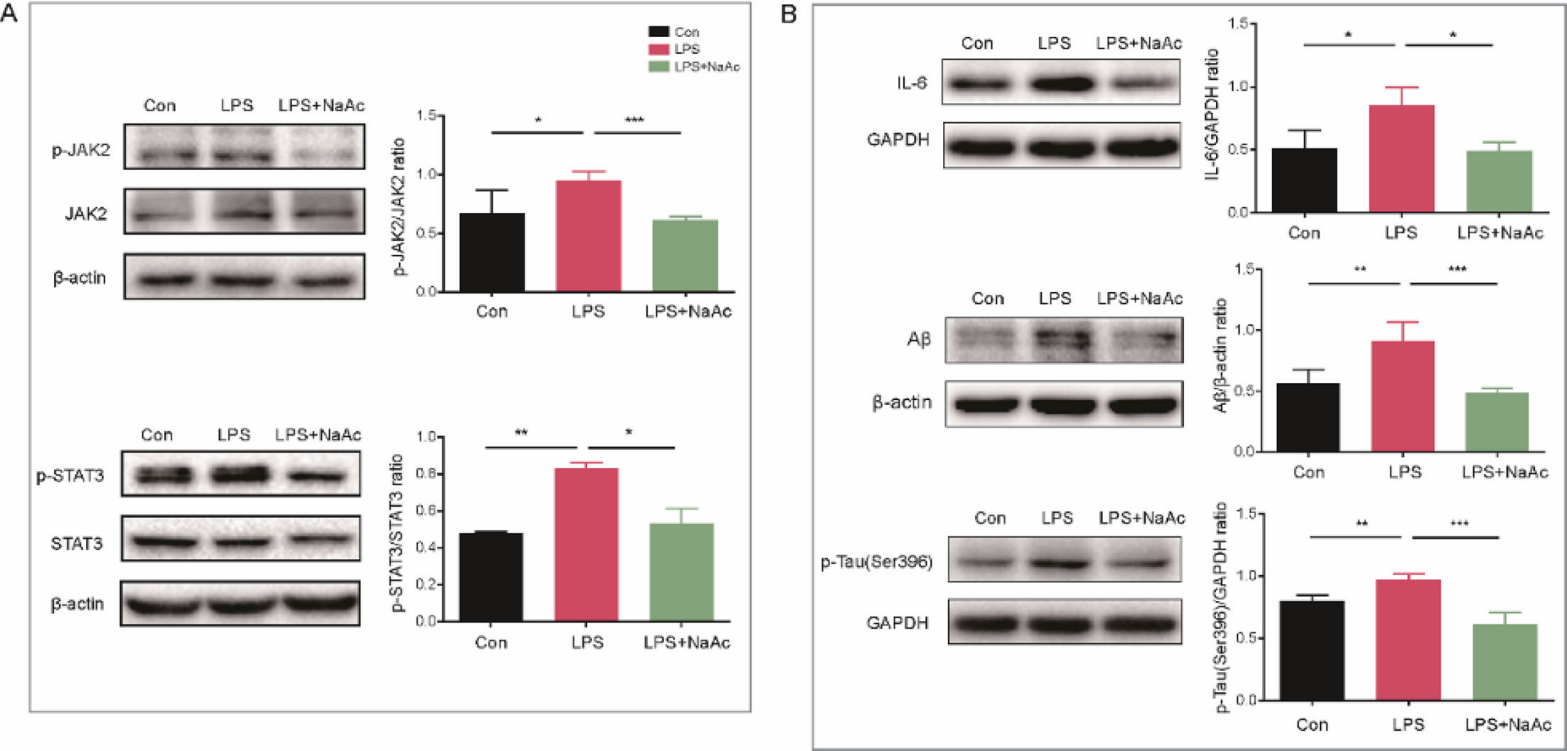
Sodium acetate inhibited microglia neuropathology by suppressing the IL-6-mediated activation of the JAK2/STAT3 signaling pathway. (**A**) Graph of expression levels and statistical differences of proteins related to the JAK2/STAT3 signaling pathway (*n* = 3 for each group). (**B**) Graph of expression levels and statistical differences of IL-6, as well as Aβ and p-Tau proteins (*n* = 3 for each group). All data were analyzed by one-way ANOVA, followed by Tukey’s multiple-comparison test. Data are expressed as mean ± SD. *P < 0.05; **P < 0.01; ***P < 0.001.

To explore the mechanism of sodium acetate, we used western blot analysis to validate the JAK/STAT3 signaling pathway, which was predicted to be involved based on transcriptome results. The data showed that the p-JAK level was significantly higher in the LPS group compared to the control group (*P* < 0.05), indicating LPS-induced JAK phosphorylation. Additionally, the p-STAT3 level was significantly elevated (*P* < 0.01), reflecting the activation of STAT3 phosphorylation. Sodium acetate treatment significantly reduced the phosphorylation levels of both proteins (*P* < 0.001 and *P* < 0.05, respectively) (Fig. 12A), indicating inhibition of the JAK/STAT3 pathway. Sodium acetate also decreased IL-6 expression levels in LPS-treated microglia (*P* < 0.001). Furthermore, levels of Aβ and p-Tau proteins, key AD pathological features, were significantly reduced following sodium acetate treatment (*P* < 0.05) (Fig. 12B). These results suggest that sodium acetate mitigates the AD pathology by inhibiting the IL-6-mediated activation of the JAK/STAT3 signaling pathway in BV2 cells.

## DISCUSSION

The findings from previous studies have suggested that probiotic interventions can positively influence AD by modulating the gut microbiota. For example, the administration of *Akkermansia muciniphila* to APP/PS1 mice has been shown to improve abnormal intestinal flora, intestinal barrier dysfunction, and dyslipidemia in AD model mice (29). Our study corroborates these observations, showing that CBM588 significantly upregulated the expression levels of intestinal barrier-related proteins and improved the pathological morphology of the colon in APP/PS1 mice (Fig. 4A and B). However, the effect of CBM588 on the overall structure of the gut microbiota was not prominent (Fig. 7A through C). This suggests that the effect of CBM588 treatment on the gut microbiota was not reflected in the change of gut bacterial diversity, but focused on the proportion of specific bacterial species. Our study also revealed that CBM588 significantly reduced inflammatory factors in both colon and brain tissues (Fig. 4C and D). This suggests that its mechanism of action on AD progression may involve modulation of inflammatory pathways through the microbiome-gut-brain axis. Neuroinflammation and systemic inflammation are known to accelerate AD progression by promoting neuronal damage and pathology. The gut microbiota plays a crucial role in regulating inflammation, and its dysregulation can impact AD pathology through inflammatory pathways mediated by components, such as LPS, amyloid peptides, bacterial metabolites (e.g., short-chain fatty acids and branched-chain amino acids), and functional byproducts (e.g., bile acids) (30–32). Microbial fragments like LPS and microbial-derived amyloids have been detected in the brain tissue of AD patients, indicating a potential link between gut microbiota and AD pathology (33). Our study found an increased abundance of inflammation-associated bacterial taxa in the intestines of APP/PS1 mice compared to the WT group (Fig. 7E and F). This included an increase in the phylum *Bacteroides*, which contains many pathogenic bacteria, and specific pro-inflammatory genera, such as *Bacteroides*, *Parasutterella*, and *Desulfovibrio* (34, 35). These gram-negative bacteria are significant as they produce LPS, which can trigger intestinal inflammation and disrupt barrier function. This disruption can lead to the entry of microbiome-derived LPS into the circulatory system and its subsequent crossing of the blood-brain barrier, contributing to chronic neuroinflammation and AD pathology. Our study also showed significantly higher expression levels of inflammatory factors in the brain of APP/PS1 mice compared to the WT group (Fig. 4D) likely due to the entry of gut-derived LPS into the brain. Additionally, the increased expression of apoptotic proteins Bax/Bcl-2 (Fig. 6B) and observed neuronal damage and apoptosis (Fig. 6A) further support the role of LPS-induced neuroinflammation and neurotoxicity in the AD pathology. In summary, pro-inflammatory flora-derived LPS in APP/PS1 mice appears to damage the intestinal barrier and contribute to AD progression through the neurotoxic and pro-inflammatory effects mediated by the microbiome-gut-brain axis.

Our study also observed a reduction in SCFA-producing bacteria in the intestines of APP/PS1 mice, particularly those producing acetic acid, such as *Alistipes* and *Acetatifactor* (Fig. 7E and F). However, following intervention with CBM588, while overall bacterial phyla levels did not significantly change, there was a notable increase in acetic acid-producing bacteria, including *Acetatifactor*, *Lactobacillus*, and *Eubacterium* (Fig. 8). This increase in acetic acid-producing bacteria was consistent with the results of targeted SCFA assays, which showed elevated levels of various SCFAs, particularly acetic acid, following CBM588 intervention (Fig. 9A and B). This finding contrasts with some previous studies (18), suggesting that the effectiveness of probiotics may vary depending on the specific strain and experimental conditions.

Although CBM588 is often associated with butyric acid production and its presumed beneficial effects through gut microbiota modulation (36), our study did not observe significant differences in butyric acid content following CBM588 intervention. This discrepancy may be due to CBM588 being a "transitory bacterium" in the intestinal tract, meaning it exerts transient microecological effects without proliferating in the gut. Emerging research suggests that the effectiveness of probiotics may depend not only on their ability to colonize but also on their capacity to share genes and metabolites, regulate dysbiotic microbiota, and influence cellular functions (37). Additionally, metabolite "cross-feeding" between bacteria, where the end products of one bacterial species' metabolism serve as substrates for another, can influence the overall metabolite profile of the gut (38, 39). The significant increase in acetic acid observed may thus be attributed to both the upregulation of acetic acid-producing bacteria and potential consumption of butyric acid by other gut bacteria.

Acetate, a key SCFA derived from the microbiome, can traverse the blood-brain barrier and impact the brain function (40). Our study found elevated levels of acetic acid in brain tissues following CBM588 intervention in APP/PS1 mice (Fig. 9C), while butyric acid levels remained below detection. This suggests that the effects of CBM588 on brain tissues are mediated predominantly by acetic acid. Research has shown that chronic acetate deficiency leads to reductions in hippocampal synaptophysin (SYP) levels and cognitive deficits, while exogenous acetate supplementation restores SYP levels and ameliorates cognitive deficits in type 1 diabetic mice (41). This highlights the potential of acetate in mitigating cognitive deficits, although the precise mechanisms remain to be fully elucidated.

Microglia, the resident immune cells of the central nervous system (CNS), are crucial for maintaining CNS homeostasis and are influenced by gut microbiota (42, 43). SCFAs, particularly acetate, play a role in microglial maturation and function. Given the role of immune cells in mediating gut microbiome-induced alterations in brain amyloid-beta (Aβ) deposition (44), targeting signaling pathways within immune cells in the brain may offer therapeutic avenues for AD (45). Our transcriptomic analysis revealed significant enrichment of differential gene expression in pathways related to the immune and digestive systems following CBM588 intervention (Fig. 10). Notably, the JAK/STAT signaling pathway was significantly enriched, suggesting its potential role in mediating the improvement of the AD pathology through acetic acid intervention. STAT3 inactivation has been implicated in AD pathogenesis (46), and alterations in JAK/STAT signaling have been associated with AD (47). This pathway is, thus, a promising target for therapeutic interventions in AD.

Our study also demonstrated that sodium acetate treatment reduced key pathological markers of AD, including Aβ and p-Tau proteins, in microglia subjected to LPS-induced inflammation (Fig. 12). Sodium acetate treatment also decreased IL-6 protein levels and phosphorylated JAK and STAT3, indicating its potential to mitigate neuroinflammation and neuronal apoptosis (Fig. 11). These findings suggest that targeting the JAK/STAT pathway and using sodium acetate as a treatment could offer new therapeutic strategies for neurodegenerative diseases, including AD.

**Fig 13:**
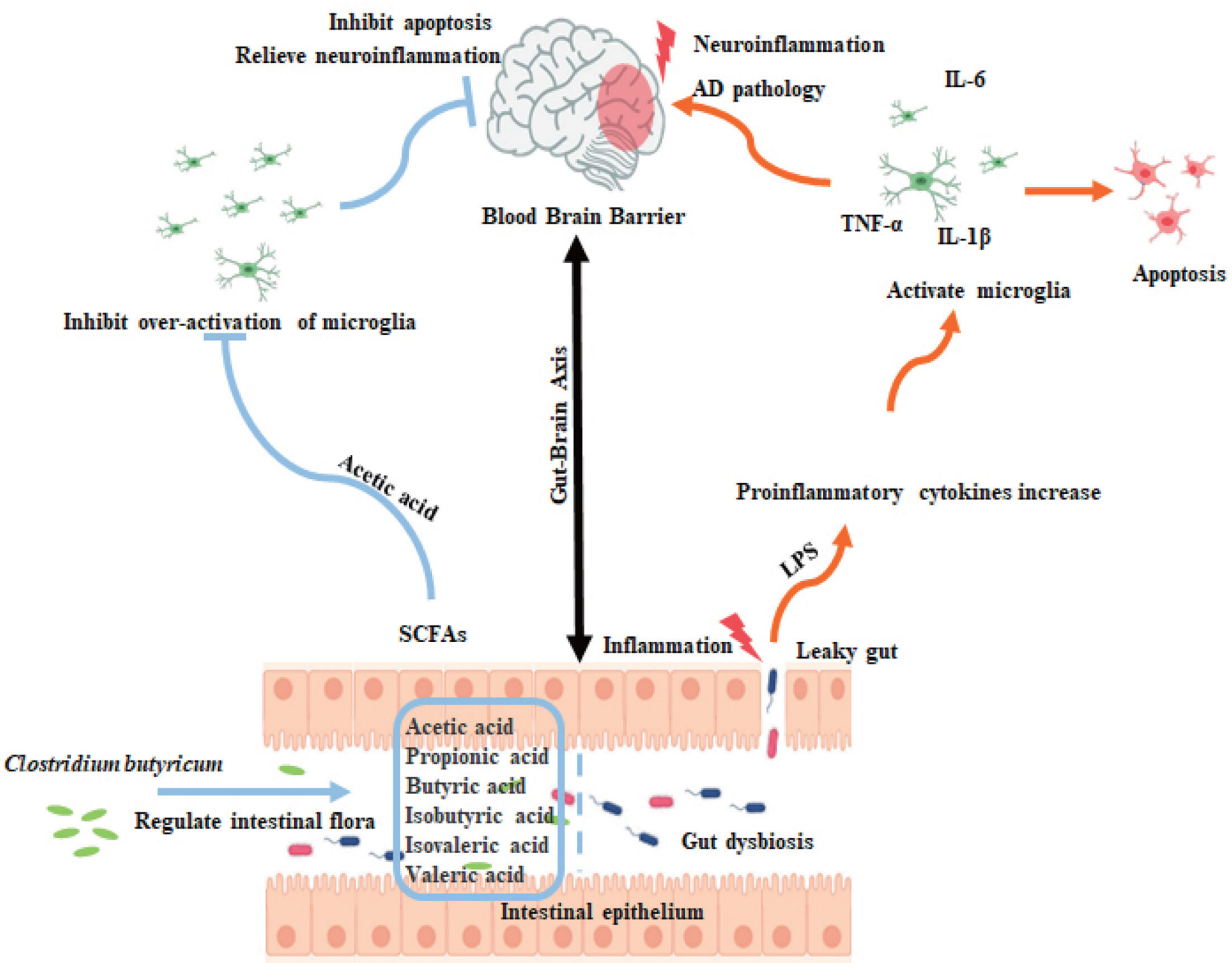
CBM588 improves cognitive impairment in APP/PS1 mice by inhibiting neuropathology and regulating the intestinal microbiota derivative acetic acid.

In conclusion, our study provides insights into the interplay between gut microbiota, neuroinflammation, and cognitive decline in AD (Fig. 13). The therapeutic potential of CBM588 represents a promising approach for addressing the multifaceted nature of AD pathology. Our findings highlight the importance of gut microbiota modulation and SCFA production in neurodegenerative diseases and pave the way for future research and clinical applications in the treatment of AD.

## Data Availability

The datasets presented in this study can be found in online repositories. The names of the repository/repositories and accession number(s) can be found below: National Center for Biotechnology Information (NCBI) BioProject, https://www.ncbi.nlm. nih.gov/bioproject/, PRJNA1063877; National Center for Biotechnology Information (NCBI) SRA, https://www.ncbi.nlm.nih.gov/sra/, PRJNA1237997 and PRJNA1244615; and National Genomics Data Center (NGDC) China National Center for Bioinformation (CNCB)/Beijing Institute of Genomics (BIG), Chinese Academy of Sciences (CAS) Open Archive for Miscellaneous Data (OMIX), https://ngdc.cncb.ac.cn/omix/, PRJCA022815 and PRJCA022816.

## SUPPLEMENTAL MATERIAL

Supplemental material spectrum.00178-25-s0001.docx Fig. S1; Table S1.

ASM does not own the copyrights to Supplemental Material that may be linked to, or accessed through, an article. The authors have granted ASM a non-exclusive, world-wide license to publish the Supplemental Material files. Please contact the corresponding author directly for reuse.
