# Supplementary material for: Clostridium butyricum enhances cognitive function in APP/PS1 mice by modulating neuropathology and regulating acetic acid levels in the gut microbiota
Source: Microbiol Spectr. 2025 Jul 7;13(8):e00178-25. doi: 10.1128/spectrum.00178-25 (PMC12323352; doi:10.1128/spectrum.00178-25)
Supplement: Supplemental material — Fig. S1; Table S1. [file spectrum.00178-25-s0001.docx]

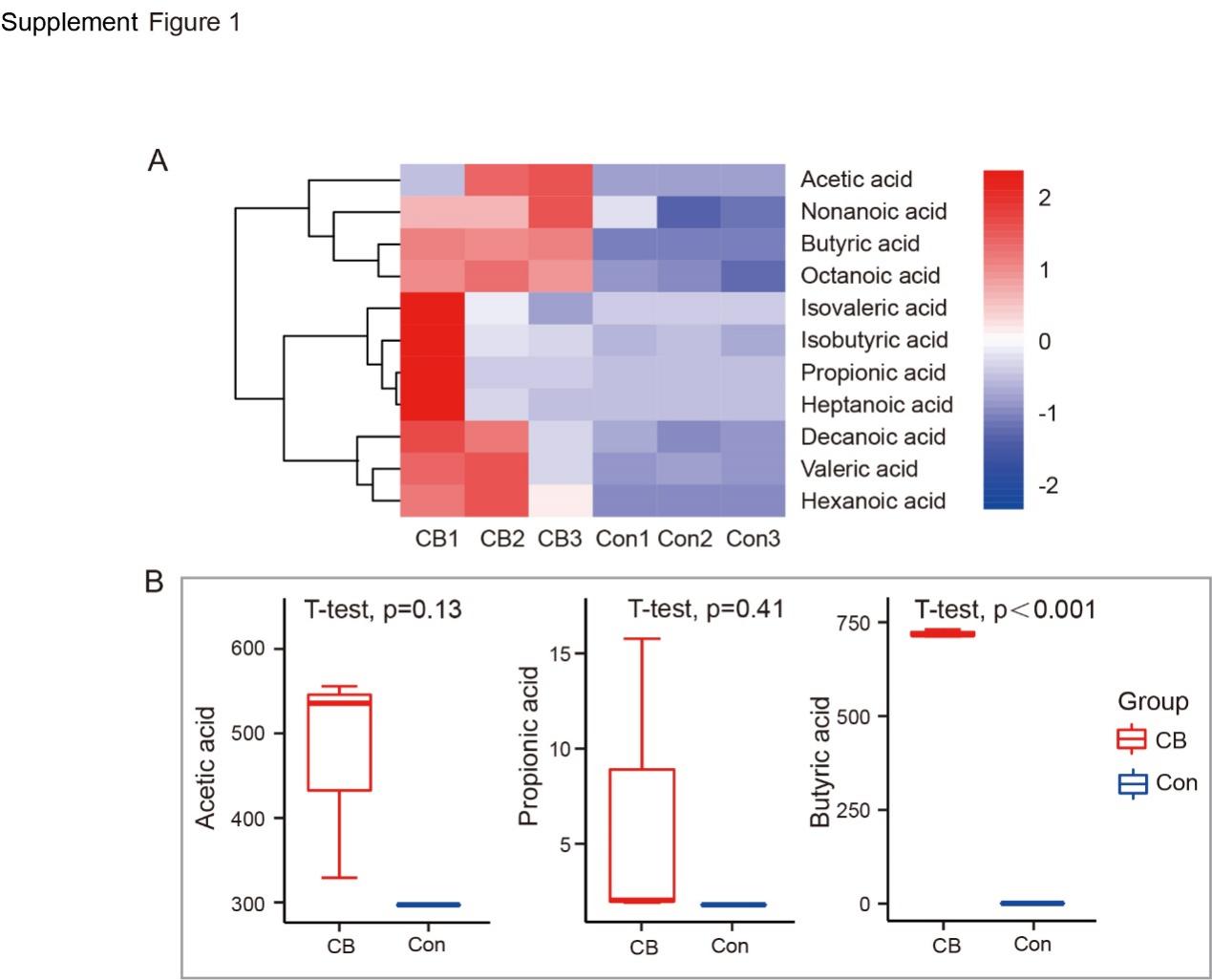


**Supplementary figure 1. Levels of short-chain fatty acid production by *Clostridium butyricum*.** (A) Heat map of short-chain fatty acid content of CBM588 culture supernatant (CB) and RCM medium control (Con), 3 samples in each group, red indicates high expression, blue indicates low expression, and the color shades represent the degree. (B) Significant difference plots for acetic, propionic, and butyric acids, with the CB group in red and the Con group in blue.****P*<0.001.

Supplement Table 1: RT-PCR specific primer sequences.

| Primer name | Accession number | Primer Sequence |
| --- | --- | --- |
| IL-6 | NM_031168 | 5′-AGGATACCACTCCCAACAGACC-3′(sense) |
|  |  | 5′-AAGTGCATCATCGTTCATACA-3′(antisense) |
| IL-1β | NM_008361 | 5′-AATGCCACCTTTTGACAGTGAT-3′(sense) |
|  |  | 5′-TGCTGCGGGATTTGAAGCTG-3′(antisense) |
| TNF-α | NM_013693 | 5′-CACGTCGTAGCAAACCACC-3′(sense) |
|  |  | 5′-TGAGATCCATGCCGTTGGC-3′(antisense) |
| β-actin | NM_007393 | 5′-GCTGTGCTATGTTGCTCTAG-3′(sense) |
|  |  | 5′-CGCTCGTTGCCAATAGTG-3′(antisense) |
